# WeCare oral kit outperforms artificial saliva in salivary flow and oral health

**DOI:** 10.3389/froh.2026.1869907

**Published:** 2026-06-23

**Authors:** Leticia Mello Bezinelli, Marcella Ferreira Gobbi, Gabriela Vitoria Carvalho Campos, Jéssica Andrade-Silva, Vitor Ulisses Monnaka, Fernanda de Paula Eduardo

**Affiliations:** 1 Hospital Israelita Albert Einstein, São Paulo, Brazil; 2 Faculdade Israelita de Ciências da Saúde Albert Einstein, São Paulo, Brazil; 3 Faculdade de Odontologia, Universidade de São Paulo, São Paulo, Brazil; 4 Department of Innovation, Hospital Israelita Albert Einstein, São Paulo, Brazil

**Keywords:** artificial saliva, intensive care units, oral hygiene, salivation, xerostomia

## Abstract

**Introduction:**

Critically ill patients frequently develop xerostomia and hyposalivation due to clinical instability and medical interventions. These conditions impair oral health, increase discomfort, and may contribute to infectious complications. Although artificial saliva is commonly used to reduce oral dryness, no standardized oral care strategy has been established for this population.

**Aims:**

To compare the effectiveness of a structured oral care intervention (WeCare oral care kit) with artificial saliva in improving salivary function, oral health, and oral comfort in patients hospitalized in intensive care and step-down units.

**Methods:**

This randomized clinical trial included 82 patients allocated to the WeCare group (*n* = 44) or the artificial saliva group (*n* = 38). Outcomes were assessed at baseline (T0) and after five oral care interventions (T1). Primary outcomes included salivary volume and flow, oral dryness, oral comfort assessed by a six-domain questionnaire, and oral health status evaluated using the Bedside Oral Exam (BOE).

**Results:**

The artificial saliva group demonstrated limited benefits, with significant improvements only in oral dryness (*p* = 0.028) and psychological comfort (*p* = 0.039). The WeCare group exhibited significant improvements in salivary volume and flow, oral dryness, and total BOE score (*p* < 0.001 for all), with improvements in five of eight BOE domains. Oral comfort improved in five of six questionnaire domains (domains 1–3 and 5–6).

**Conclusions:**

The WeCare oral care kit provided broader and more consistent clinical benefits than artificial saliva, demonstrating superior effectiveness in improving salivary function, oral health, and patient-reported oral comfort.

**Clinical Trial Registration:**

ClinicalTrials.gov, NCT07555444, https://clinicaltrials.gov/study/NCT07555444

## 1 Introduction

The oral care of patients hospitalized in Intensive Care Units (ICUs) or Step-Down Units (SDUs) is often compromised due to their debilitated condition (1). Moreover, these patients frequently present xerostomia, hyposalivation, and lip dryness as consequences of pharmacological treatments and mechanical ventilation (2, 3). These factors contribute to the development of several oral complications, including speech impairment, oropharyngeal dysphagia, and discomfort (4).

Beyond compromising patient well-being, impaired oral health also increases the risk of local and pulmonary infections (4). Saliva exerts both mechanical and immunological functions that inhibit the growth of pathogenic bacteria in the oral cavity (5). In patients with hyposalivation, bacterial load rises, favoring the formation of dental biofilm, whose accumulation progresses with the length of hospitalization (6). In addition to elevating the risk of local infections, this bacterial overload facilitates aspiration into the pulmonary tract (5, 6), which represents a major cause of ventilator-associated pneumonia (VAP), a condition associated with high mortality rates and substantial treatment costs.

The importance of a structured oral care routine for ICU patients was highlighted by a 2003 study reporting up to a 60% reduction in the incidence of VAP (7). Notably, expenses related to oral care represent less than 10% of the costs associated with treating nosocomial pneumonia (6), underscoring the cost-effectiveness of such strategies. Examples of measures implemented to improve oral care in hospitalized patients include rinsing the oral cavity with chlorhexidine solution, manual or electric toothbrushing, and mechanical cleaning of teeth and gums with gauzes (8). Artificial saliva is widely used due to its recognized role in maintaining oral health (9). Nevertheless, no gold-standard method for oral care in ICU patients has been established.

The WeCare company produces an oral care kit that includes a mouthwash for oral hygiene, used as an oral rinse for 30 s, followed by the application of the oralcare gel (2 g sachet), which is spread throughout the oral mucosa and teeth. The formulations contain compounds associated with oral hydration, including xylitol (present in both formulations) and hyaluronic acid (present in the oralcare gel) (10–12). The present study evaluated the effects of this oral care protocol on salivary flow, oral health, and oral health–related quality of life in ICU patients, in comparison with the conventional use of artificial saliva.

## 2 Materials and methods

### 2.1 Study design and setting

This randomized controlled trial (RCT) was conducted in the intensive care and step-down units of the Einstein Hospital Israelita between October 2022 and January 2024, after ethical clearance had been granted by the Institutional Ethics Committee (approval number 5.622.861). Throughout the study, all ethical principles, practical guidelines, and biosafety regulations established by the Brazilian Ministry of Health and the institutional Human Research Ethics Committees were strictly followed. Written informed consent was obtained from all participants prior to enrollment. The trial compared the performance of two distinct oral hygiene and lip hydration protocols. Participants were allocated into two parallel arms, receiving either the WeCare Oral Care Kit (WeCare group) or compounded artificial saliva as per the hospital's standard protocol (Artificial saliva group). The primary outcome of the study was unstimulated salivary flow. Secondary outcomes included degrees of oral dryness, oral health according to Bedside Oral Exam (BOE) scores, and oral comfort. The trial was registered retrospectively at ClinicalTrials.gov (identifier: NCT07555444).

### 2.2 Sample size and criteria

Based on the institutional incidence of 41% traumatic oral lesions among the 234 patients who had been intubated in 2021, and considering a medium effect size of 0.438, a sample size of 130 patients, evenly divided between the two groups, was calculated to ensure a statistical power of 0.8. Eligible participants were adult between 18 and 69 years, admitted to either the ICU or SDU, who had at least four teeth present in the oral cavity and showed signs of dryness in the oral mucosa or lips. Patients were excluded if they had been admitted following scheduled surgery, presented infectious foci or lesions in the oral cavity, had sustained trauma to the oral cavity, had incomplete or inconsistent medical records, were using any type of topical oral solution, were unconscious, or had been hospitalized due to COVID-19.

### 2.3 Randomization

Participants were randomly assigned in a 1:1 ratio to WeCare group or Artifical saliva group using a manual simple randomization procedure performed by an independent researcher who was not involved in data collection or clinical intervention. Allocation was performed without stratification or blocking. To ensure unpredictability, the study team generated a sequence of assignments using a manually prepared set of opaque, sequentially numbered slips (each indicating one of the two study arms), which were shuffled thoroughly before enrollment began. For each participant, the next slip in the sequence was drawn and the assignment recorded. No member of the clinical team had access to the remaining allocation slips during the enrollment process, minimizing the risk of selection bias.

### 2.4 Intervention

All participants underwent the same basic oral hygiene routine, consisting of daily toothbrushing with fluoridated toothpaste. Interventions were performed every 12 h, immediately after the hospital's standard oral hygiene procedure. In Artificial saliva group, patients rinsed with water (4 or 10 mL) for 30 s. Subsequently, the healthcare professional provided a 2 g sachet of artificial saliva, instructing the patient to spread the product throughout the oral mucosa by moving the tongue over all areas. In WeCare group, following the same initial hygiene routine, patients rinsed with the WeCare Oral Care Kit mouthwash (5 or 10 mL) for 30 s and then applied the kit's oral gel (2 g sachet), spreading it over the entire oral mucosa and teeth. After the application, patients in both arms were advised to refrain from eating or drinking for 20 min and received standardized oral care instructions from the Hospital Dentistry Team.

### 2.5 Formulations of oral care interventions

The mouthwash manufactured by WeCare consisted of aqua, sorbitol, glycerin, hydroxyethylcellulose, sodium benzoate, potassium sorbate, glutamine, sodium fluoride, xylitol, aroma, and PEG-40 hydrogenated castor oil.

The oralcare gel manufactured by WeCare consisted of aqua, glycerin, hydroxyethylcellulose, sodium benzoate, potassium sorbate, glutamine, hydrolyzed hyaluronic acid, and xylitol.

The artificial saliva, compounded by the hospital pharmacy department, consisted of potassium chloride 0.096%, sodium chloride 0.067%, magnesium chloride 0.004%, calcium chloride 0.012%, potassium phosphate 0.027%, and purified water added to a final volume of 100 mL.

### 2.6 Data collection

**Figure 1:**
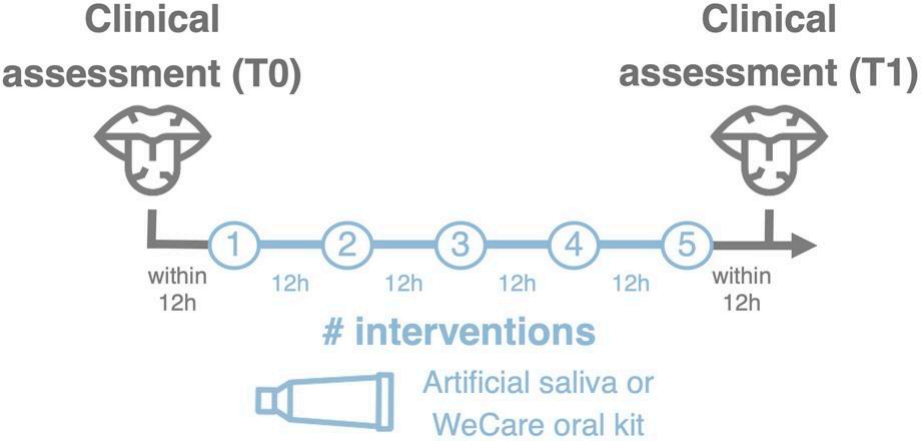
Study timeline and intervention schedule. Clinical assessments were conducted before the first intervention (T0) and after the fifth intervention (T1). Assessments were performed within 12 h of the corresponding intervention sessions. Participants received five intervention sessions at 12-hour intervals, resulting in approximately 60 h of product use.

Clinical assessments were conducted by the same hospital dentist, who remained blinded to group allocation throughout the study and followed standardized protocols to ensure consistency across measurements. Assessments were performed within 12 h of the corresponding intervention sessions, before the first intervention (T0) and after the fifth intervention (T1), resulting in approximately 60 h of product use, as illustrated in Figure 1.

The degree of dryness of the oral mucosa was classified on a 0–4 scale, assessed visually under standardized lighting, with patients positioned supine and the bed inclined at 30–45°.

Oral health conditions were evaluated using the BOE scale (10), which comprises eight-clinical criteria (swallowing, lips, tongue, saliva, mucous membranes, gums, teeth or dentures, and odor), each scored from 1 to 3. Higher cumulative scores reflected worse oral health status, with a score of 1 indicating normal condition and a score of 3 indicating severe impairment. The total score ranges from 8 to 24 points, with 8 points indicating good oral health and 24 points representing severe dysfunction. Salivary flow rate (mL/min) was calculated from the total unstimulated salivary volume expectorated over a standardized three-minute period into a sterile graduated 5 mL syringe, with the collected volume measured directly in milliliters using the syringe graduations.

An oral-comfort questionnaire was administered at both T0 and T1 immediately following the corresponding clinical evaluation. Domains related to general psychological well-being and social aspects of oral health, typically included in instruments such as the OHIP-14, were not included because participants were admitted to the ICU or SDU, where these factors are inherently influenced by clinical status. To reduce cognitive load and enhance comprehensibility in patients potentially experiencing mild cognitive impairment or fatigue, a five-point visual Likert scale with schematic facial expressions was used to represent increasing levels of positivity (1–5 points). To minimize participant burden, the questionnaire was kept brief and comprised six domains (Supplementary Table S1): psychological comfort, physical comfort, functional comfort, functional limitation, dryness sensation, and physical pain.

Demographic characteristics (age and sex) and admission diagnoses were collected retrospectively from electronic medical records.

### 2.7 Statistical analysis

Statistical analyses were performed using R software (version 4.1.1), adopting a significance level of 5%. Normality of quantitative variables was assessed using the Shapiro–Wilk test. Comparisons of quantitative variables between study arms were performed using the Mann–Whitney U test, whereas categorical variables, including sex and admission diagnoses, were compared using the chi-square test or Fisher's exact test, as appropriate. Within-arm comparisons across time points were conducted using the Wilcoxon signed-rank test. For paired qualitative variables, marginal homogeneity tests were applied to evaluate changes in ordinal categorical outcomes.

For ordinal outcomes that improved in both treatment groups, cumulative link mixed models (CLMMs) were applied to test whether the magnitude of change across time points differed between the artificial saliva and WeCare interventions. Results are reported as model coefficients ± standard error, test statistic, and *p*-value. Coefficients represent log-odds of cumulative probabilities, where negative values indicate a higher likelihood of lower outcome categories.

## 3 Results

### 3.1 Study population

**Figure 2:**
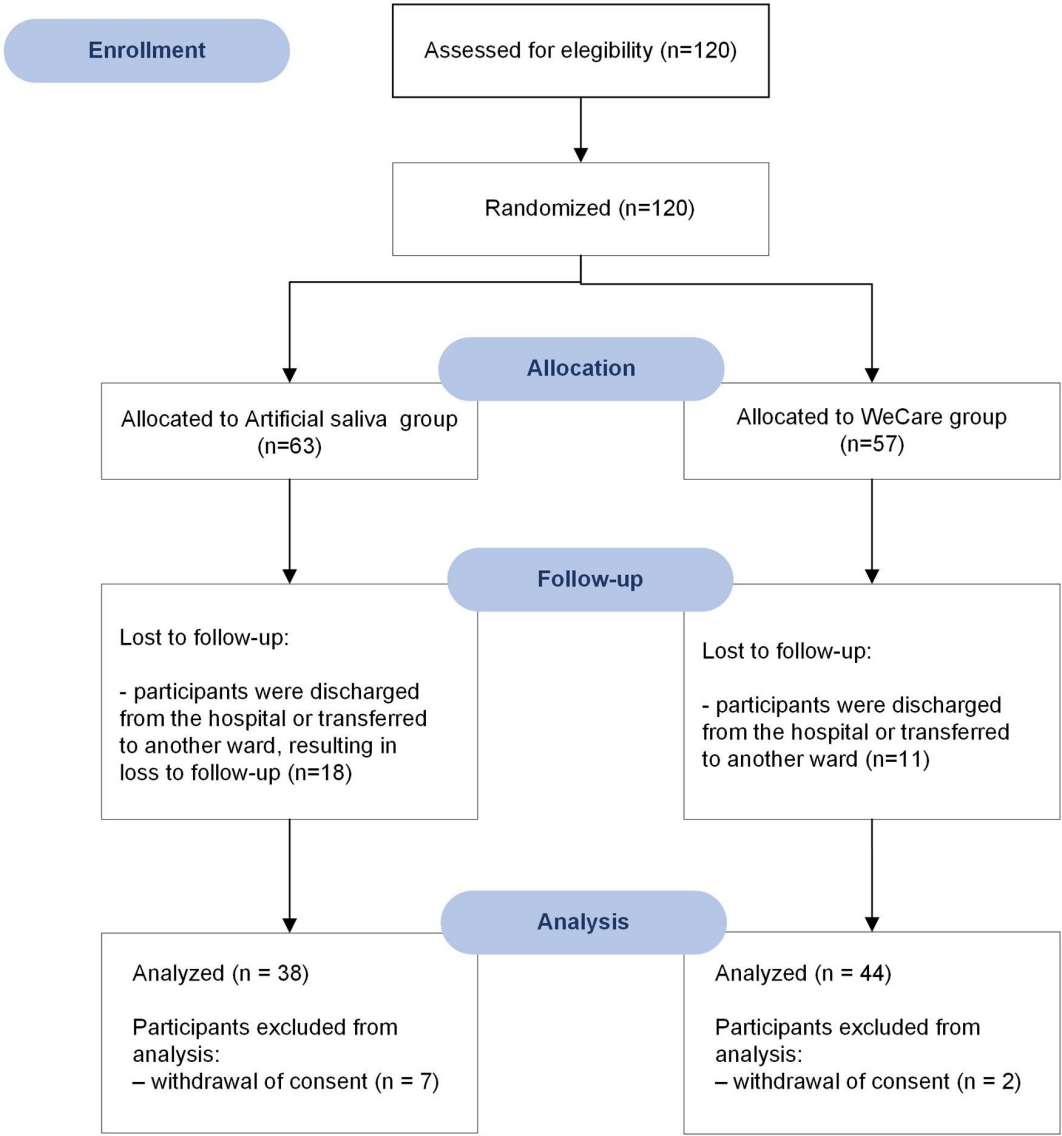
Study flow diagram.

From 120 patients enrolled in the study, 63 were allocated to the Artificial saliva group and 57 to the WeCare group. During the follow-up period, 29 participants were excluded due to hospital discharge or transfer to another ward, resulting in loss to follow-up. In addition, 9 participants withdrew consent during the analysis phase. Completed data were therefore available for 82 patients, comprising 38 in the Artificial saliva and 44 in the WeCare intervention group (Figure 2). The comparable proportion of losses to follow-up between groups minimizes the risk of differential attrition bias. Baseline sociodemographic characteristics and admission diagnoses are presented in Table 1. The study population consisted predominantly of male patients, with no significant between-group differences in age or sex distribution. Common admission diagnoses included sepsis, heart failure, chronic kidney disease, and pneumonia. Admission diagnoses represented by a single patient were grouped under the category “Others” to facilitate data presentation. Although most diagnoses were similarly distributed between groups, chronic kidney disease was more frequent in the WeCare group (18.2% vs. 0.0%; *p* = 0.020).

**Table 1:** Sociodemographic characteristics and admission diagnoses of the study groups.

Characteristic	Artificial saliva (*n* = 38)	WeCare (*n* = 44)	*P* value
Sociodemographic characteristics			
Age (years)	49.8 (39.3, 62.8)	53.4 (44.0, 63.5)	0.139*
Male sex	26 (68.4)	29 (65.9)	0.995^†^
Admission diagnosis			
Sepsis	9 (23.7)	12 (27.3)	0.722^†^
Heart failure	2 (5.3)	6 (13.6)	0.696^‡^
Chronic kidney disease	0 (0.0)	8 (18.2)	0.020^‡^
Pneumonia	5 (13.2)	2 (4.5)	0.090^‡^
Acute myocarditis	1 (2.6)	2 (4.5)	1.000^‡^
Essential hypertension	1 (2.6)	2 (4.5)	1.000^‡^
Atrial fibrillation	2 (5.3)	0 (0.0)	0.136^‡^
Disruption of operation wound	0 (0.0)	2 (4.5)	0.523^‡^
Stroke	1 (2.6)	1 (2.3)	1.000^‡^
Others	17 (44.7)	9 (20.5)	

Continuous variables are reported as median (Q1, Q3), and categorical variables are presented as counts (percentages).

Admission diagnoses represented by a single patient were grouped under the category “Others”.

* Mann–Whitney test.

† Chi-squared test.

‡ Fisher's exact test.

### 3.2 Salivary volume and flow

**Figure 3:**
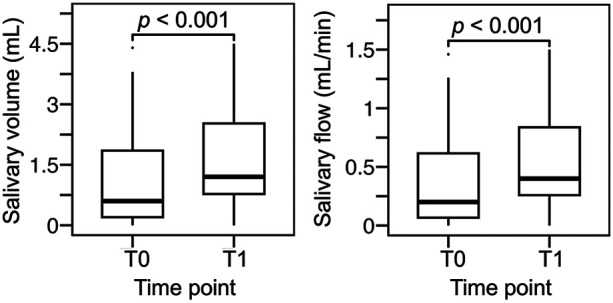
Increased salivary secretion following the WeCare intervention. Boxplots show salivary volume and salivary flow at baseline (T0) and post-intervention (T1) in the WeCare group. Both parameters increased significantly after the intervention (Wilcoxon test).

At baseline (T0), no significant differences were observed between the Artificial saliva and WeCare groups in either salivary volume or salivary flow (Supplementary Table S2). Likewise, at post-intervention (T1), both parameters remained comparable between groups. Within-group analyses showed no significant changes in the Artificial saliva group from T0 to T1 (*p* = 0.525 for volume; *p* = 0.568 for flow; Supplementary Figure S1). In contrast, the WeCare group exhibited significant increases in both salivary volume and flow after the intervention (*p* < 0.001 for both; Figure 3).

An exploratory mixed-effects analysis was performed to evaluate the influence of sex on salivary flow. Overall, men exhibited higher salivary flow values than women (estimated marginal mean: 0.516 vs. 0.325 mL/min, respectively; *p* = 0.014). However, no significant interaction between treatment group, time, and sex was observed (*p* = 0.377), indicating that the effect of the intervention was comparable between sexes.

### 3.3 Degree of oral dryness

**Figure 4:**
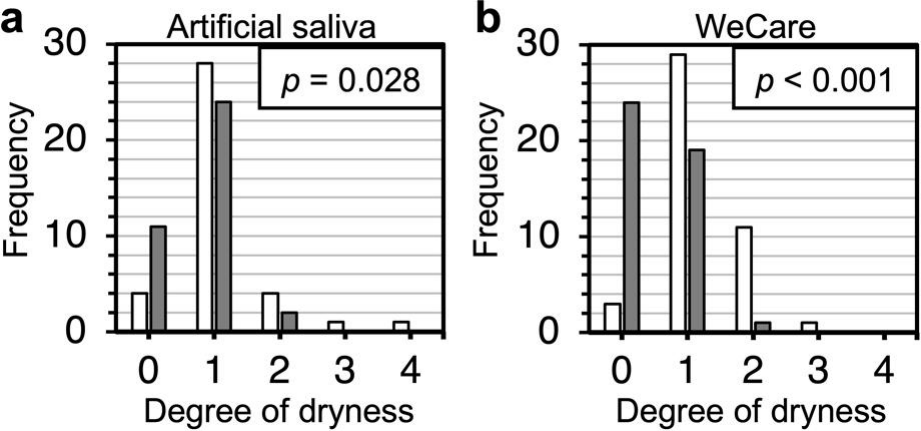
Degree of oral dryness at baseline (T0) and post-intervention (T1) in artificial saliva and WeCare groups. Bar charts show the frequency distribution of dryness degrees in **(a)** Artificial saliva and **(b)** WeCare groups. Both groups exhibited improvement after interventions (marginal homogeneity test).

At baseline (T0), the distribution of dryness degrees was comparable between the Artificial saliva and WeCare groups (*p* = 0.342). However, at post-intervention (T1), the WeCare group exhibited a lower proportion of patients in the higher oral dryness categories (*p* = 0.050). Within-group comparisons from T0 to T1 showed significant improvements in both Artificial saliva (*p* = 0.028; Figure 4A) and WeCare (*p* < 0.001; Figure 4B) groups. Cumulative link mixed model (CLMM) analysis confirmed a significant interaction between group and time points, indicating that the WeCare group showed a greater reduction in dryness compared with the Artificial saliva group (*β* = −2.125 ± 0.766, z = −2.738, *p* = 0.006).

### 3.4 Oral health

The Bedside Oral Exam (BOE) scale was used to evaluate overall oral health, with higher scores indicating poorer oral status. No significant differences were observed between groups at baseline (T0; *p* = 0.066) or after the intervention period (*p* = 0.137) in total BOE scores, as shown in Supplementary Table S3. Within-group analyses showed that BOE scores did not significantly change in the Artificial saliva group (*p* = 0.105), whereas the WeCare group exhibited a marked reduction from T0 to T1 (median 10 vs. 8; *p* < 0.001), indicating improvements in oral health condition.

**Figure 5:**
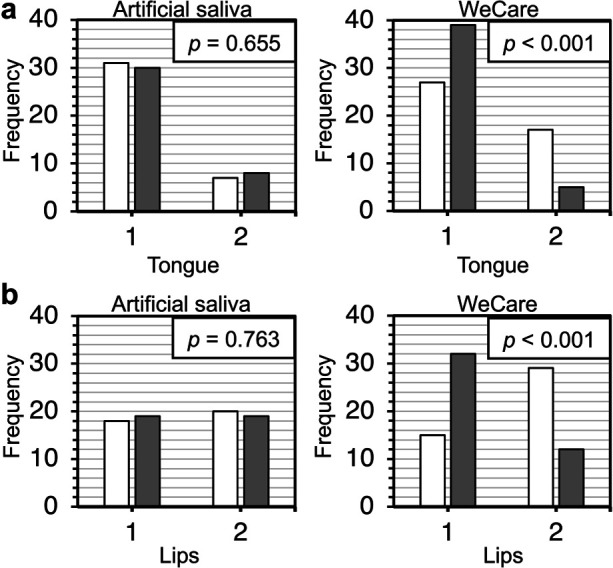
Changes in tongue and lip conditions according to the bedside oral exam (BOE) scale. Frequency distributions of tongue **(a)** and lip **(b)** conditions at baseline (T0) and post-intervention (T1) in the Artificial saliva (left) and WeCare (right) groups. The Artificial saliva group showed no significant changes after the intervention, whereas the WeCare group exhibited marked improvements in both tongue and lip conditions (marginal homogeneity test).

**Figure 6:**
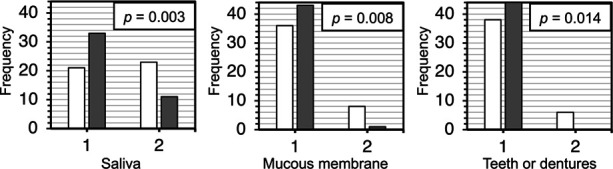
Additional improvements in bedside oral exam (BOE) parameters in the WeCare group. Frequency distributions of saliva, mucous membrane, and teeth or dentures conditions at baseline (T0) and post-intervention (T1) in the WeCare group. Significant improvements were observed for all parameters after the intervention (marginal homogeneity test).

In the comparison of individual BOE items between groups (Supplementary Table S4), WeCare patients exhibited worse tongue conditions at baseline (more patients in class 2; *p* = 0.045), while differences for lips were no statistically significant (*p* = 0.222). After the intervention (T1; Supplementary Table S5), differences in tongue conditions were no longer significant (*p* = 0.231), whereas WeCare participants showed more favorable lip scores (more patients in class 1; *p* = 0.034). In within-group analyses (Figure 5), the Artificial saliva group showed no improvement across any item, whereas the WeCare group presented significant improvements not only in tongue and lips (*p* < 0.001 for both; Figure 5), but also in saliva (*p* = 0.003), mucous membrane (*p* = 0.008), and teeth or dentures (*p* = 0.014; Figure 6).

### 3.5 Oral comfort

**Figure 7:**
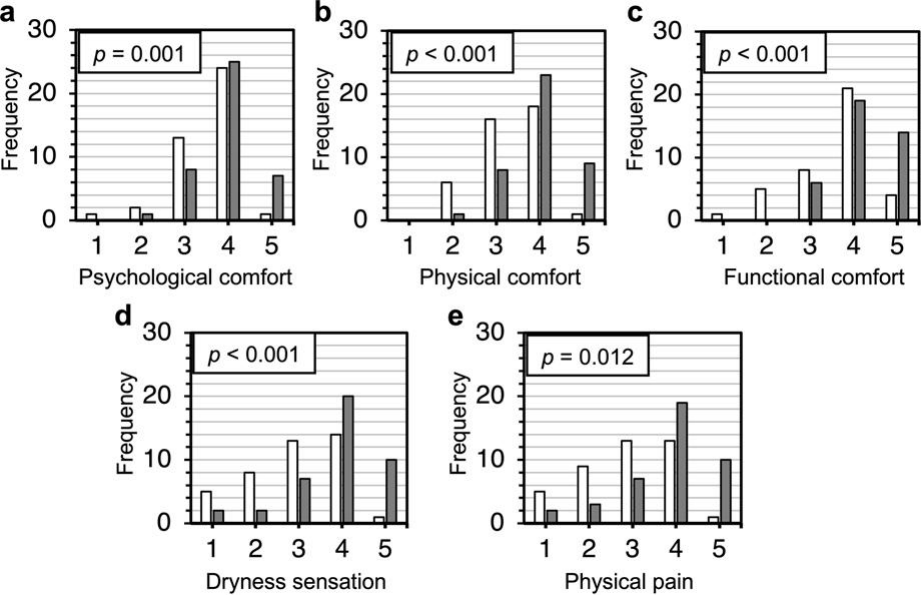
Improvements in oral comfort in the WeCare group. Frequency distributions of psychological comfort **(a)**, physical comfort **(b)**, functional comfort **(c)**, dryness sensation **(d)**, and physical pain **(e)** at baseline (T0) and post-intervention (T1) in the WeCare group. Significant improvements were observed for all items after the intervention (marginal homogeneity test).

Oral comfort was evaluated using a questionnaire described in methods section (Supplementary Table S1). At baseline (T0), participants in the WeCare group exhibited worse scores than those in the Artificial saliva group in items related to psychological comfort (*p* = 0.006), physical comfort (*p* = 0.005), functional comfort (*p* = 0.002), and dryness sensation (*p* = 0.022). After the intervention (T1), no significant differences were observed between groups. Within-group analyses revealed that the Artificial saliva group (Supplementary Figure S2) showed improvement only in psychological comfort (*p* = 0.039), whereas the WeCare group (Figure 7) exhibited significant improvements in psychological comfort (*p* = 0.001), physical comfort (*p* < 0.001), functional comfort (*p* < 0.001), dryness sensation (*p* < 0.001), and physical pain (*p* = 0.012). CLMM analysis for psychological comfort did not show a significant interaction between group and time points, indicating that the magnitude of improvement did not differ significantly between interventions (*p* = 0.454).

## 4 Discussion

In this study, we evaluate the effectiveness of a commercial oral care kit (WeCare) in improving salivary flow and oral health in hospitalized in patients hospitalized in Intensive Care Units (ICUs) and Step-Down Units, in comparison with the conventional use of artificial saliva. Although recruitment did not reach the initially estimated sample size due to the high turnover of ICU beds, statistically significant differences were detected in several outcomes.

The benefits of using artificial saliva in ICU patients were limited. No measurable differences were detected in salivary volume, salivary flow, or total BOE score. However, improvement was verified in the degree of oral dryness. Interestingly, such improvements were sufficient to positively influence patient psychological comfort, suggesting that even partial hydration of the oral cavity can have a perceptible impact on patient comfort.

The heterogeneous response to artificial saliva observed in this study appears to partially align with a report evaluating the effect of Mucosamin® artificial saliva spray in COVID-19 patients under non-invasive mechanical ventilation, which showed variable improvements in Beck oral assessment scale (BOE) scores, with marked gains on the first day followed by a decline in the next two days (13). Consistent with our findings, artificial saliva administration did not improve salivary flow in diabetic patients with xerostomia, despite improving xerostomia scores from 2.9 to 1.4 in ambulatory settings (14). As highlighted by other studies (3), critically ill ICU patients are exposed to multiple complex risk factors for xerostomia, such as polypharmacy, mechanical ventilation, reduced oral intake, and systemic inflammation, which could have limited both the durability and magnitude of the response to artificial saliva.

In contrast, the WeCare group exhibited substantial improvements across multiple oral health outcomes. Before the intervention, this group presented higher frequence of tongue dryness (worse BOE score), a baseline difference that may be partially related to the higher frequency of chronic kidney disease in the WeCare group, a condition commonly associated with xerostomia and salivary alterations (15). As a consequence, these patients also reported a greater negative impact of oral health on their well-being, as measured by the oral comfort questionnaire, especially in domains related to psychological comfort, physical comfort, functional comfort, and dryness sensation. After the intervention, salivary flow and volume increased, leading to a significant reduction in oral dryness. The total BOE score also ameliorated, with notable gains in the subdomains of lips, saliva, tongue, mucous membranes, and teeth or dentures. Importantly, the WeCare group demonstrated a greater reduction in oral dryness compared with the Artificial saliva group. Additionally, the initial disadvantage in tongue dryness was no longer present, and fewer patients present oral dryness and lip dryness in this group. These findings suggest that the WeCare kit not only mitigated the worse baseline conditions of these patients but also provided clinical benefits beyond those achieved with artificial saliva.

These effects may be partially explained by the hydrating and mucosal-protective properties of the formulation components. Hyaluronic acid, present in the oralcare gel, may contribute to the formation of a protective moisture-retaining layer over the oral mucosa, helping preserve hydration and maintain a favorable environment for epithelial integrity and tissue repair (10). In parallel, xylitol, present in both the mouthwash and oralcare gel, has been associated with stimulation of salivary secretion through mechanical, gustatory, and parasympathetic reflex pathways, potentially contributing to the increased salivary flow observed in the WeCare group (11, 12). Additionally, symptom relief may have been partially attributable to glycerin, a humectant present in both formulations that promotes moisture retention and oral lubrication. Similar improvements in xerostomia-related symptoms, including difficulties with swallowing and speaking, have been reported following the use of glycerol-based saliva substitutes such as Aequasyal® oral spray (Carilène Labora-tory, Montesson, France), supporting the role of glycerin-containing formulations in alleviating oral dryness and improving patient comfort (16, 17). Together, these mechanisms may also contribute to reducing lip dryness by improving overall hydration of the oral mucosa and increasing salivary volume.

Oral dryness and impaired salivary defense mechanisms are well-established risk factors for bacterial overgrowth and biofilm accumulation in the oral cavity (6), which increases the risk of developing ventilator-associated pneumonia (VAP), a major cause of mortality among ICU patients (5). Accordingly, the improvements in hydration related to the use of the WeCare kit itself may help prevent bacterial proliferation in the oral cavity. In addition, the oral hygiene promoted by the mouthwash included in this kit may contribute to reducing pathogenic bacterial load through oral rinsing, despite the absence of antiseptic agents in its formulation, which are commonly associated with increased oral dryness and mucosal irritation (18). Although this study was not designed to assess infection-related outcomes, future trials can explore the impact of the WeCare kit in reducing the incidence of VAP or other nosocomial infections in comparison with antiseptic strategies, which should include evaluation of potential adverse effects like xerostomia and mucosal irritation.

Importantly, the oral health–related quality of life of the intervention group improved across several domains, including psychological comfort, physical comfort, functional comfort, dryness sensation, and physical pain. Although improvements in psychological comfort were similar between groups, these findings highlight the more comprehensive impact of the WeCare kit relative to artificial saliva. From a clinical perspective, the observed improvements are highly relevant. As core aspects of patient well-being, even modest gains in functional limitation, physical pain, and psychological discomfort may translate into meaningful improvements in overall quality of life for critically ill patients. Collectively, these results emphasize that interventions targeting oral dryness and discomfort should be regarded as integral components of patient-centered care in ICUs.

This study has several limitations that should be considered. Although the trial was originally powered for 130 participants, recruitment was limited by the high turnover of intensive care units’ beds, resulting in a smaller final sample and potentially reduced power for detecting subtle between-group differences in secondary outcomes. Randomization without stratification led to baseline imbalances in oral health status and oral comfort, with worse initial conditions in the WeCare group; however, this group still demonstrated greater improvement after the intervention. Some outcomes relied on subjective or semi-quantitative assessments, such as visual grading of oral dryness and self-reported comfort, which may be influenced by observer or response bias despite the use of validated instruments. Additionally, improvements in oral comfort may have been partially influenced by clinical recovery or participant awareness of study participation. However, significant differences between groups were observed despite both groups being exposed to the same follow-up schedule and assessment procedures. Salivary flow estimation may have been influenced by the relatively short collection period. However, the same standardized collection protocol was applied to both groups under identical conditions, reducing the likelihood of systematic bias between interventions. Finally, although improved oral hydration and hygiene may reduce bacterial load and ventilator-associated pneumonia risk, this study was not designed to evaluate infection-related outcomes. Despite these limitations, the findings provide clinically meaningful evidence supporting the benefits of the WeCare oral care kit over artificial saliva in critically ill patients and inform future research directions.

## Data Availability

The raw data supporting the conclusions of this article will be made available by the authors, without undue reservation.
