# Supplementary material for: WeCare oral kit outperforms artificial saliva in salivary flow and oral health
Source: Front Oral Health. 2026 Jun 23;7:1869907. doi: 10.3389/froh.2026.1869907 (PMC13337760; doi:10.3389/froh.2026.1869907)
Supplement: Supplementary file 1 [file Datasheet1.pdf]

Supplementary Material

WeCare oral kit outperforms artificial saliva in salivary flow and oral health

1 Supplementary Figures

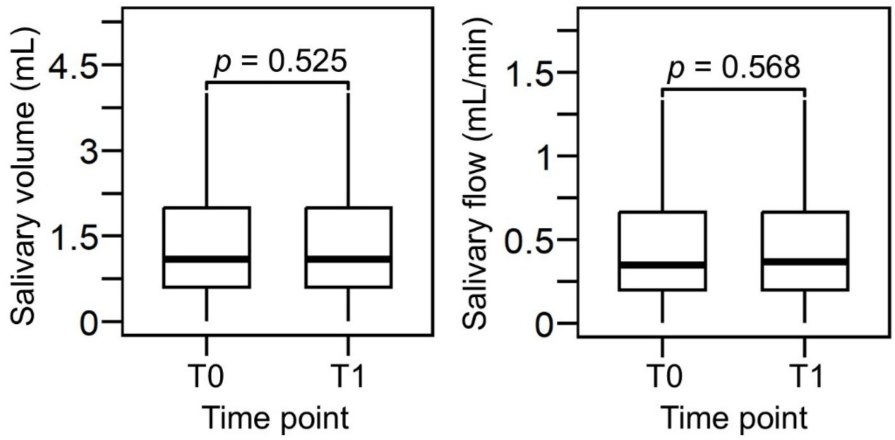

Supplementary Figure 1. Unchanged salivary parameters in the Artificial saliva group. Boxplots show salivary volume and salivary flow at baseline (T0) and post-intervention (T1) in the Artificial saliva group. No significant differences were observed between time points (Wilcoxon test).

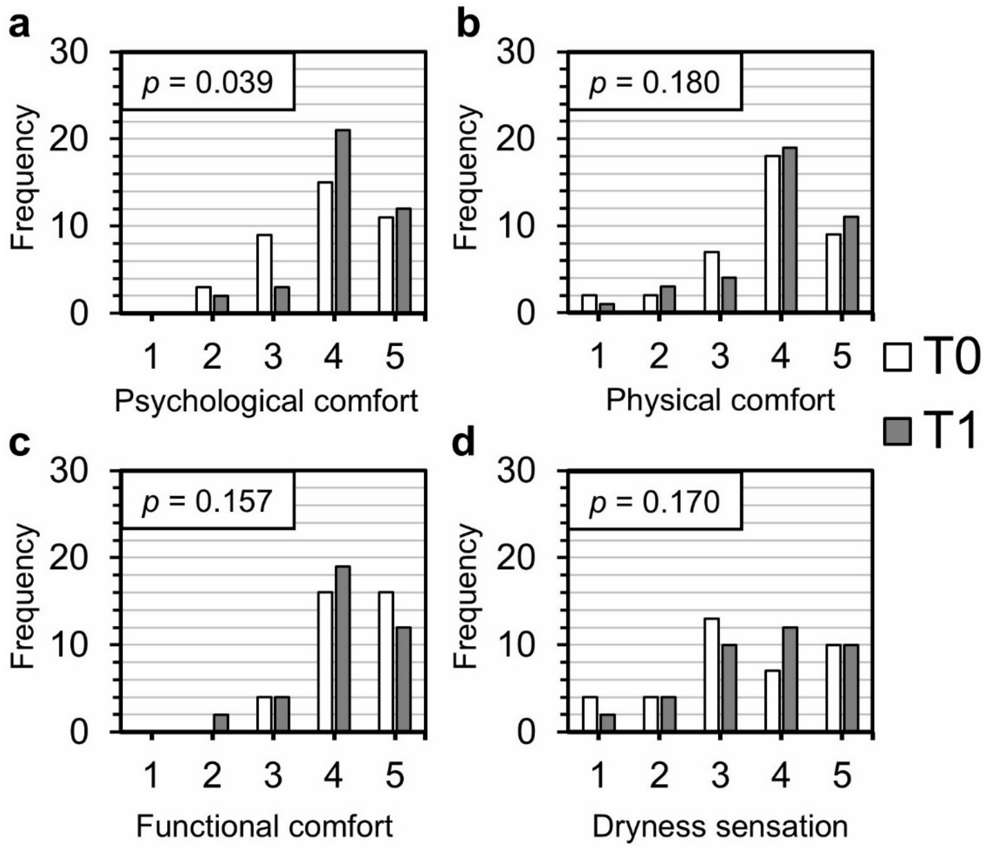

Supplementary Figure 2. Changes in oral comfort in the Artificial saliva group. Frequency distributions of psychological comfort (a), physical comfort (b), functional comfort (c), and dryness sensation (d) at baseline (T0) and post-intervention (T1) in the Artificial saliva group. Significant improvements were only observed for Q1 (marginal homogeneity test).

2 Supplementary Tables

Domain	Question
Psychological comfort	How are you feeling today?
Physical comfort	How comfortable is your mouth?
Functional comfort	How comfortable is eating?
Functional limitation	If you had discomfort, did it interfere with eating?
Dryness sensation	How dry is your mouth?
Physical pain	Have you experienced pain in your mouth?

Supplementary Table 1. Oral-comfort questionnaire.

Variables	Time point	Artificial saliva	WeCare	*p* value
Salivary volume (mL)	T0	1.1 (0.6, 2)	0.6 (0.2, 1.85)	0.103
	T1	1.1 (0.6, 2)	1.2 (0.78, 2.52)	0.305
Salivary flow (mL/min)	T0	0.35 (0.2, 0.67)	0.2 (0.07, 0.62)	0.129
	T1	0.37 (0.2, 0.66)	0.4 (0.26, 0.84)	0.342

Supplementary Table 2. Salivary volume and flow (mL/min) at baseline (T0) and post-intervention (T1) in the Artificial saliva and WeCare groups. Values are presented as median (Q1, Q3). Statistical comparisons were performed using the Mann-Whitney test.

Time point	Artificial saliva	WeCare	*p* value†
T0	9 (9, 10)	10 (9, 11)	0.066
T1	9 (8, 10)	8 (8, 9)	0.137
p* value	0.105	<0.001	-

Supplementary Table 3. Bedside Oral Exam (BOE) scores at baseline (T0) and post-intervention (T1) in the Artificial saliva and WeCare groups. Values are presented as median (Q1, Q3). Statistical analysis was performed using the Mann-Whitney test for comparison between groups† and the Wilcoxon test for comparison between time points*.

Variable	Score	Artificial saliva (n=38)	WeCare (n=44)	*p* value
Swallowing	1	37 (97.4)	38 (86.4)	0.278‡
	2	0 (0)	3 (6.8)	
	3	1 (2.6)	3 (6.8)	
Lips	1	18 (47.4)	15 (34)	0.222†
	2	20 (52.6)	29 (65.9)	
Tongue	1	31 (81.6)	27 (61.4)	0.045†
	2	7 (18.4)	17 (38.6)	
Saliva	1	20 (52.6)	21 (47.7)	0.577‡
	2	17 (44.7)	23 (52.3)	
	3	1 (2.6)	0 (0)	
Mucous membranes	1	32 (84.2)	36 (81.8)	0.999‡
	2	6 (15.8)	8 (18.2)	
Gums	1	37 (97.4)	44 (100)	0.463‡
	2	1 (2.6)	0 (0)	
Teeth or dentures	1	35 (92.1)	38 (86.4)	0.494‡
	2	3 (7.9)	6 (13.6)	
Odor	1	36 (94.7)	43 (97.7)	0.594‡
	2	2 (5.3)	1 (2.3)	

Supplementary Table 4. Bedside Oral Exam (BOE) scores in the study groups before intervention (T0). Variables are presented as counts (percentages). †Chi-squared test, ‡Fisher's exact test.

Variable	Score	Artificial saliva (n=38)	WeCare (n=44)	*p* value
Swallowing	1	37 (97.4)	39 (88.6)	0.430‡
	2	0 (0)	3 (6.8)	
	3	1 (2.6)	2 (4.5)	
Lips	1	19 (50)	32 (72.7)	0.034†
	2	19 (50)	12 (27.3)	
Tongue	1	30 (78.9)	39 (86.6)	0.231†
	2	8 (21.0)	5 (11.4)	
Saliva	1	26 (68.4)	33 (75)	0.618‡
	2	11 (28.9)	11 (25)	
	3	1 (2.6)	0 (0)	
Mucous membranes	1	35 (92.1)	43 (97.7)	0.332‡
	2	3 (7.9)	1 (2.3)	
Gums	1	38 (100)	44 (100)	-
	2	0 (0)	0 (0)	
Teeth or dentures	1	36 (94.7)	44 (100)	0.212‡
	2	2 (5.3)	0 (0)	
Odor	1	37 (97.4)	44 (100)	0.463‡
	2	1 (2.6)	0 (0)	

Supplementary Table 5. Bedside Oral Exam (BOE) scores in the study groups after intervention (T1). Variables are presented as counts (percentages). †Chi-squared test, ‡Fisher's exact test.
